# Current News

**Published:** 1896-05

**Authors:** 


### Current News.

### THE GOLDEN ANNIVERSARY.

The dentists of Philadelphia and vicinity met at the Continental Hotel on December 16, 1895, to celebrate with a banquet, the fiftieth anniversary of the formation of the first dental society, and the first successful movement for the promotion of dental education in Pennsylvania.

Professor James Truman presided, while on his left sat the venerable Dr. Spencer Roberts, who is one of the three surviving original members of that association.

The banquet was prepared and served in excellent style, and at its close the president, calling to order, briefly alluded to the reasons that induced the profession in Philadelphia to celebrate this occasion, and called upon Dr. William H. Trueman, who gave an historical sketch of the formation of the original society, "The Pennsylvania Association of Dental Surgeons," in which he said that just fifty years ago, on December 16, 1845, that society met for the first time as an organized body, in the lecture-room of the Philadelphia Museum, a building that occupied about the spot where they were then assembled. This Association was established for a specific purpose; it was the outcome of an earnest desire of the progressive dentists of Philadelphia to establish in this city a dental college, having a strong State dental society at its back, and they were impressed that it would be more in touch with the profession and its possible usefulness thereby increased.

For several years these matters have been talked of at social gatherings, prominent in which were Drs. John DeHaven White, Elisha Townsend, E. B. Gardette, Samuel L. Mintzer, Lewis Roper, Robert Arthur, and others.

The Pennsylvania Association of Dental Surgeons held its first meeting with the following officers: President, Dr. Gustavus A. Plantou; Vice-President, Dr. Stephen T. Beale; Recording Secretary, Dr. C. C. Williams; Corresponding Secretary, Dr. Robert Arthur; Treasurer, Dr. F. Reinstein.

Referring to this Association, the "History of Oral and Dental Science in America" has this to say, "This was the first organization which did not, almost immediately upon its formation, express its reprobation of amalgam; in fact, its whole constitution and by-laws breathe a spirit of tolerance and primal regard for the science over any outside issues, which is in some contrast to its predecessors in existence. It has probably done more real work, quietly and without ostentation, than any other dental society."

The speaker then said that after the society was fully organized renewed efforts were made to establish a college. In this, however, unlooked for opposition was encountered, so that it was not until October, 1852, that the long-wished-for, long-hoped-for dental college was opened by Dr. Elisha Townsend, who delivered the introductory lecture.

Dr. Trueman then gave a brief history of the profession, and closed by saying that the gathering in pleasant harmony of the dental societies and the colleges of Philadelphia at this festive board was to honor the memory of those who fifty years ago did for us so much, and did it so well.

Professor C. N. Peirce was then called upon, and he spoke at some length upon the establishment of systematic dental education in Philadelphia. The early struggles of the few earnest and courageous men were reviewed. How the charter for the Pennsylvania College of Dental Surgery was secured, the college starting in a small way, occupying the upper stories of the building on Arch Street near Sixth, then known as the Jones, White & McCurdy building, the predecessors of the S. S. White Dental Manufacturing Company. From this they moved to Tenth and Arch Streets, and some years later, as the demands increased, to a larger building at Twelfth and Filbert Streets, and three years ago the faculty was again forced to look for larger accommodations. They then built and removed to their new building, northeast corner of Eleventh and Clinton Streets, below Spruce Street. The first faculty of this institution was composed of Elisha Townsend, Robert Arthur, J. F. B. Flagg, Eli Parry, and Thomas L. Buckingham. A remarkable array of talent for that stage of dental education.

The Pennsylvania College of Dental Surgery, from the time of its organization to the present, the close of the last session, has conferred the degree of Doctor of Dental Surgery upon eighteen hundred and twenty-five students, sixty of whom have been women.

The president then called upon Professor S. H. Guilford, who gave a review of the establishment and growth of the Philadelphia Dental College. He spoke especially and with much feeling of the services and life of its late dean, Professor James E. Garretson. This speaker was followed by Professor Edwin T. Darby, who gave, in a few well-chosen words, an historical sketch of the birth, growth, and present position in the dental world, of the dental department of the University of Pennsylvania.

Among the others who spoke were Dr. Charles F. Bonsall, the present president of the Association, and Dr. Jesse C. Green, of West Chester, who gave a number of his interesting reminiscences. Dr. J. D. Thomas then favored the assemblage with a well-rendered vocal selection.

There was no address of the evening listened to with more appreciative attention than that of Professor Wilbur F. Litch, who was introduced as the editor of that monumental work,—one of the greatest landmarks in dental education,—"The American System of Dentistry." He said, in part, "In listening, as I have, with absorbing interest to the history of this organization and to the reminiscences connected therewith, I have been impressed with the thought that as all social organizations are made up of members drawn together by some common bond of comradeship, some special tie, either of taste, of interest, or of vocation, each organized body takes its individuality from the dominant qualities of those who frame it, and each may be regarded as a composite of its members, a composite which represents character.

"Character to the social organizer is what the soil is to the seed, it determines development, growth, fruitage.

"Many of those who organized this Association I knew personally, and knowing them can realize how much of force and earnestness and zeal and fidelity and courage there was back of the movement in which their hearts were enlisted, and to which they gave so much of the best effort of their lives. There was character back of the Association, and that is why it has lived and why, once formed, it became a social factor as well as an educational power.

"As a social factor, how much it must have accomplished in quieting antagonism, harmonizing misunderstandings, and overcoming prejudices. The younger generation of dentists can hardly realize to what extent jealousy and mistrust obtained among those who had few if any opportunities of really meeting upon common ground, of gauging the worth and merit each of the other. It is difficult to overestimate the value of such organizations in unifying professional sentiment, the first and essential step to true professional advancement.

"There are to-day in Philadephia, in addition to several dental societies, three large educational institutions, while throughout the land similar organizations have multiplied.

"What the achievements of the past half-century have been all know; still greater results may be looked for in the next century so soon to open. To-night we turn a retrospective gaze in the fifty years of honor and usefulness through which the Pennsylvania Association of Dental Surgeons has lived. In honor and usefulness may she still live when a century hence the breath of that newer cycle shall fan the brows of those who, following us, shall then meet to do her honor."

The festivities were then closed by the president, who alluded to the gratification that this celebration had afforded him, and in the retrospective glance given, let us not forget, as we separate, to also labor for the future with the hope that the incoming century would show a far larger and broader development.

G. W. W.

---

### AMERICAN MEDICAL ASSOCIATION.

### PROGRAMME FOR THE DENTAL AND ORAL SECTION.

"Chairman's Address." Dr. R. R. Andrews, Cambridge, Mass.

"A Few Causes of Failures in the Dental and Medical Professions."
Dr. B. B. Smith, Pensacola, Fla.

"Modern Methods of treating the Antrum of Highmore."
Dr. W. Xavier Sudduth, Chicago, Ill.

"Further Investigations upon the Antrum."
Dr. M. H. Fletcher, Cincinnati, Ohio.

"Cataphoresis." Dr. H. W. Gillett, Newport, R. I.

"The Technique and Pathology of the Peridental Membrane."
Dr. V. A. Latham, Chicago, Ill.

"Movements of the Mandibular Condyles and Dental Articulation."
Dr. W. E. Walker, Pass Christian, Miss.

"Disease of the Oral Cavity—a Patent Factor of General Disease."
Dr. S. W. Foster, Atlanta, Ga.

"Treatment of Children during the Period of Dentition."
Dr. H. H. Johnson, Macon, Ga.

"Professional Congeniality." Dr. H. D. Wilson, Bainbridge, Ga.

"The Replacement of the Superior Maxilla by a Mechanical Appliance."
Dr. Thomas P. Hinman, Atlanta, Ga.

"Practical Illustrations in Conservative Surgery."
Dr. G. Lenox Curtis, New York City.

Title not yet received. Dr. G. V. I. Brown, Duluth, Minn.

"Pyorrhœa Alveolaris. Dr. E. S. Talbot, Chicago, Ill.

### LOUISIANA STATE DENTAL SOCIETY.

THE following list comprises the recently-elected officers of the Louisiana State Dental Society, and that of the officers and members of the Board of Dental Examiners:

President, Dr. J. H. Landry, Plaquemine, Iberville Parish, La.; First Vice-President, Dr. R. L. Zelinka; Second Vice-President, Dr. M. W. Rainold; Recording Secretary, Dr. C. V. Vignes; Corresponding Secretary, Dr. S. J. Bourgeois, Morgan City; Treasurer, Dr. L. D. Archinard.

*Executive Committee.*—Dr. J. J. Sarrazin, chairman, 828 Canal Street, New Orleans; Dr. C. Mermilliod, Sr., Dr. John W. Adams, Dr. C. Ratsburg, Dr. A. J. Foret; Secretary, Dr. Wallace Wood, 621 Canal Street, New Orleans.

*Board of Dental Examiners.*—President, Dr. A. G. Friedrichs, 641 St. Charles Street, New Orleans; Dr. M. R. Fisher, Dr. J. M. Comegys, Dr. L. D. Archinard; Secretary, Dr. J. Paul Bayon, 643 Bourbon Street, New Orleans.

*Special Fund Committee.*—Dr. A. L. Plough, chairman, 1039 Canal Street, New Orleans; Dr. J. J. Sarrazin; Treasurer, Dr. C. V. Vignes, cor. Canal and Bourbon Streets, New Orleans.

The Society will hold its next meeting in New Orleans, La., on the 3d of March, 1897, the day following Mardi Gras.

C. V. VIGNES,
*Secretary.*

---

### DENTAL SOCIETY OF THE STATE OF NEW YORK.

THE Twenty-eighth Annual meeting of the above Society will be held in Geological Hall, Albany, on the 13th and 14th of May, 1896, to which the profession is invited.

The following is the programme:

### PROGRAMME.

"Pyorrhœa Alveolaris,—Its Causation, Diagnosis, and Treatment." C. N. Peirce, D.D.S., Philadelphia.

"Professional Fees." S. G. Perry, D.D.S., New York.

"The Application of Medicaments in Pathological Conditions of the Oral Cavity." M. W. Foster, M.D., D.D.S., Baltimore.

"The Latest Achievement in Dental Art." R. Ottolengui, M.D.S., New York.

"Report of Committee on Practice." M. L. Rhein, M.D., D.D.S., New York.

H. J. BURKHART, *President.*

BUFFALO. CHARLES S. BUTLER, *Secretary.*

### NEW ORLEANS ACADEMY OF STOMATOLOGY.

THE following-named officers were elected by the New Orleans Academy of Stomatology for the ensuing year:

President, Dr. Louis D. Archinard; Vice-President, Dr. Mozart W. Rainold; Secretary and Treasurer, Dr. C. V. Vignes.

The Academy meets on the fourth Wednesday of each month.

C. V. VIGNES,
*Secretary.*

